# Support mechanisms for research generation and application for postgraduate students in four universities in Uganda

**DOI:** 10.1186/s12961-021-00776-0

**Published:** 2021-09-15

**Authors:** E. A. Obuku, R. Apunyo, G. Mbabazi, D. K. Mafigiri, C. Karamagi, F. Sengooba, J. N. Lavis, N. K. Sewankambo

**Affiliations:** 1 Clinical Epidemiology Unit, Department of Medicine, School of Medicine, College of Health Sciences, Makerere University, P.O. Box 7072, Kampala, Uganda; 2 McMaster Health Forum, Department of Health Research Methods, Evidence and Impact, and Department of Political Science, McMaster University, Hamilton, Canada; 3 Department of Health Policy and Planning, School of Public Health, College of Health Sciences, Makerere University, Kampala, Uganda; 4 Department of Social Work and Social Administration, College of Humanities and Social Sciences, Makerere University, Kampala, Uganda; 5 The Africa Centre for Systematic Reviews and Knowledge Translation, College of Health Sciences, Makerere University, Kampala, Uganda; 6 Regional East African Community Health (REACH) Policy Initiative, College of Health Sciences, Makerere University, Kampala, Uganda; 7 Africa Centre for Evidence (ACE), University of Johannesburg, Johannesburg, South Africa; 8 Center for Social Science Research On AIDS, Department of Anthropology, College of Arts and Sciences, Case Western Reserve University, Cleveland, OH USA; 9 Faculty of Epidemiology and Population Health, London School of Hygiene and Tropical Medicine, London, UK

**Keywords:** Student, Postgraduate, Research support mechanisms, Uganda

## Abstract

**Background:**

A large proportion of postgraduate students the world over complete a research thesis in partial fulfilment of their degree requirements. This study identified and evaluated support mechanisms for research generation and utilization for masters’ students in health institutions of higher learning in Uganda.

**Methods:**

This was a self-administered cross-sectional survey using a modified self-assessment tool for research institutes (m-SATORI). Postgraduate students were randomly selected from four medical or public health Ugandan universities at Makerere, Mbarara, Nkozi and Mukono and asked to circle the most appropriate response on a Likert scale from 1, where the “situation was unfavourable and/or there was a need for an intervention”, to 5, where the “situation was good or needed no intervention”. These questions were asked under four domains: the research question; knowledge production, knowledge transfer and promoting use of evidence. Mean scores of individual questions and aggregate means under the four domains were computed and then compared to identify areas of strengths and gaps that required action.

**Results:**

Most of the respondents returned their questionnaires, 185 of 258 (71.7%), and only 79 of these (42.7%) had their theses submitted for examination. The majority of the respondents were male (57.3%), married or cohabiting (58.4%), and were medical doctors (71.9%) from Makerere University (50.3%). The domain *proposal development for postgraduate research project* had the highest mean score of 3.53 out of the maximum 5. Three of the four domains scored below the mid-level domain score of 3, that is, *the situation is neither favourable nor unfavourable*. Areas requiring substantial improvements included priority-setting during *research question identification*, which had the lowest mean score of 2.12. This was followed by *promoting use of postgraduate research products*, tying at mean scores of 2.28 each. The domain *knowledge transfer of postgraduate research products* had an above-average mean score of 2.75.

**Conclusions:**

This study reports that existing research support mechanisms for postgraduate students in Uganda encourage access to supervisors and mentors during proposal development. Postgraduate students’ engagement with research users was limited in priority-setting and knowledge transfer. Since supervisors and mentors views were not captured, future follow-on research could tackle this aspect.

## Background

A large proportion of postgraduate students the world over complete a research thesis in partial fulfilment of their degree requirements [18, 22]. Typically, postgraduate students in institutions of higher learning design, conduct and report their research work, and in the process inculcate critical skills of the scientific method. Supervisors and mentors guide postgraduate students through question identification, proposal development and finding solutions to public health problems in their area of interest. The level of research project support from question identification to promoting the use of knowledge products of these theses was unknown globally, and that is why this study was necessary. The full breadth of the thesis involves a field exercise of collecting data from study participants in institutions such as the hospital or the general community before cleaning and analysis. Finally, the student writes a full research report and defends these results in a dissertation defence before being permitted to graduate [10, 16, 19]. However, these processes have not been robustly evaluated formally in Uganda.

Assessment of postgraduate students’ research support mechanisms is crucial to inform policy action at universities in Uganda and similar settings globally. More often than not, it is the students who are assessed by a panel of university dissertation examiners. Yet, in order to implement comprehensive solutions to improve the quality of the thesis process, it is vital students contribute to this assessment and point out system challenges. Previous studies of South African and Malaysian postgraduates have reported the thesis supervision and academic support, albeit did not document research to uptake for decision-making or policy change [16, 19].

Several approaches have been proposed to assess the performance of research entities. A unique one is the knowledge translation (KT) self-assessment tool for research institutes (SATORI) [13], which was designed to assess the status of KT in research organizations. Other methods have been limited to evaluating research production, commonly used as a basis for funding [7]. This performance-based funding for universities in high- and middle-income countries has been criticized for resulting in diminished returns over time [15]. This is because in performance-based funding, there are costly processes to obtain reliable and comparable information; the cutthroat competition decreases diversity by creating a shift towards so-called safe research, discouraging experiments with new approaches, regardless of whether this research benefits society. “Publication inflation” and “game-playing” without essentially improving performance whilst widening the gap between research and teaching has also been linked to performance-based funding of universities [3, 6, 29].

Therefore, the objective of this study was to identify and evaluate support mechanisms for research generation and utilization for masters’ students in health institutions of higher learning in Uganda. Support mechanisms include seminars, workshops, co-supervision and funding.

## Methods

### Design

This was a cross-sectional survey using a modified SATORI (m-SATORI) [12, 21]. The cross-sectional design provided more recent information about the existing mechanisms to conduct and apply students’ research and was feasible in terms of resource input. The SATORI was piloted among five students for face validity and content and modified to suit the Ugandan context. Prior to this study, the original SATORI was used to evaluate KT strategies in Tehran University of Medical Sciences (TUMS) [12]. It should be noted that the creators of the original instrument, Gholami and colleagues, preferred to administer this tool to a mixed group of staff at TUMS, for completion during a discussion meeting. However, in an environment comprising students and teachers, the power hierarchy is likely to compromise the responses of students [2]. Hence, students in our study responded individually.

### Setting and sampling

Four health institutions of higher learning were purposively selected. The College of Health Sciences, Makerere University (MakCHS) and Mbarara University of Science and Technology (MUST) represented the public education system. MUST is located about 250 km from Kampala city in Mbarara district, western Uganda, and provides clinical and public health postgraduate training. MUST was opened in 1989, and it was not until the late 1990s that its first postgraduate student was channelled out [23]. The two private universities were Uganda Martyrs University, Nkozi (UMU), which opened in 1993 [32], and the Uganda Christian University, Mukono (UCU), which was established in 1997 [31]. UMU is located about 100 km from the capital, whilst UCU is 30 km away. UCU and UMU were the first private universities in Uganda to provide postgraduate training in public health-related courses.

At each university, the lists of registered postgraduate students pursuing public health or medical courses was obtained. A random sample was selected in proportion to their estimated size (data not shown) using a computerized approach in an open-access website (https://www.randomizer.org/) [33]. Those who had not commenced their proposal writing process or pursued veterinary medicine were excluded.

### Sample size estimation

The sample size was computed by online open-access software OpenEpi (https://www.openepi.com/Menu/OE_Menu.htm) [9] based on the following assumptions. The average of the mean scores for KT activities at TUMS across the four domains of SATORI was (2.26 + 2.92 + 2 + 1.89)/4 = 2.27 out of 5, which was proportionally 45.4% [12]. Since the level of KT activity in Ugandan universities was unknown, it was reasonable to estimate this score at the midpoint of 50%. It was estimated that at least 1000 postgraduate students were enrolled, of whom about 300 were in advanced stages of their research projects. These variables were imputed into OpenEpi software, taking a design effect of 1.75 ± 5% precision and adjusting for 10% nonresponse; the sample size was *n*_0_ = 536. Further adjusting for a small population of those in advanced stages, *N* = 300 using Cochran’s formula *n*_1_ = *n*_0_/[1 + (*n*_0_ − 1)/*N*], the estimated required sample size for the survey, *n*_1_, was 192 participants [8].

### Data collection

This m-SATORI used a five-level Likert scale [20] to capture data. Students were instructed to circle the most appropriate response as 1 where the situation was unfavourable and/or there was a need for an intervention, and 5 where the situation was good and needed no intervention. Responses 2, 3 and 4 represented a spectrum on a sliding scale, where 3 was the midpoint and implied the situation was neither favourable nor unfavourable.

These questions were asked under four domains: (i) *the research question*—to what extent does your department support you to identify research needs of health research users and convert them into research questions? (11 questions); (ii) *knowledge production*—to what extent does your department provide support in proposal development and research conduct? (5 questions); (iii) *knowledge transfer*—do we have appropriate means for disseminating the organizations’ research results to their target audiences? (14 questions); (iv) *promoting use of evidence*—do we help decision-makers utilize research results better? (4 questions).

### Analysis and outcome measures

Proportions were used to describe the participants’ characteristics. The m-SATORI specific data was summarized into the following domains: (a) the question of research, (b) knowledge production, (c) knowledge transfer and (d) promoting use of evidence. Mean scores of individual questions and aggregate means under the four domains were computed. Mean scores were compared across the four domains to identify areas of strengths and gaps that required intervention. Regression analyses were not done.

## Results

### Characteristics of respondents

Most of the respondents returned their questionnaires, 185 of 258 (71.7%) (Table 1). A majority of the postgraduates were in the fourth decade of their lives (55.7%), married (58.4%), medics (71.9%) and from MakCHS (50.3%). In terms of the nature of their postgraduate courses, a majority by far were full-time students (84.3%), pursuing clinical courses (67%) and under self-sponsorship (68.6%). Four fifths (81.6%) had presented their proposals for approval by the local university institutional research and ethics boards, whilst only 79 (42.7%) had their theses submitted for examination.

**Table 1:** Baseline characteristics of postgraduate students at four universities in Uganda

Variable	*N* = 185 (%)	Variable	*N* = 185 (%)
Age (years)		Gender	
18–30	64 (34.6)	Female	78 (42.7)
31–40	103 (55.7)	Male	106 (57.3)
41–50+	18 (9.7)	Course nature	
Marital status		Full time	156 (84.3)
Married/cohabiting	108 (58.4)	Distance learning	29 (15.7)
Never married	74 (40)	Sponsorship status	
Separated/divorced	3 (1.6)	Self	127 (68.6)
University		Funder	58 (31.4)
MakCHS	93 (50.3)	Proposal status	
MUST	45 (24.3)	Submitted	151 (81.6)
UCU	27 (14.6)	Not submitted	34 (18.4)
UMU	20 (10.8)	Thesis status	
Professional qualification		Submitted	79 (42.7)
Medical doctor	133 (71.9)	Not submitted	106 (57.3)
Nursing/midwifery	12 (6.5)	PG degree course	
Clinical officer	11 (6)	MMED	124 (67)
Pharmacy	9 (4.8)	MPH/PPM	50 (27)
Other professions	20 (10.8)	MSc EpiBio/MHSR/Clin trials	6 (3.2)
		MSc Pharmacology	5 (2.7)

*MMed* Masters of Medicine (medicine, surgery, paediatrics, obstetrics and gynaecology), *MPH* Masters of Public Health, *MHSR* Masters Health Services Research, *MSc* Master of Science, *EpiBio* clinical epidemiology and biostatistics, *Clin trials* clinical trials, *PPM* project planning and management, *MakCHS* Makerere University College of Health Sciences, *MUST* Mbarara University of Science and Technology, *UCU* Uganda Christian University, *UMU* Uganda Martyrs University, *PG* postgraduate

### Mean score per domain of postgraduate research support activities at the department level

The domain *proposal development for postgraduate research project* had the highest mean score of 3.53 out of the maximum 5. Level 1 implied that *the situation is quite unfavourable and/or there is a dire need for intervention*, whilst at level 5, *the situation is good and needs no intervention*. Three of the four domains scored below the mid-level domain score of 3, that is, *the situation is neither favourable nor unfavourable* (Table 2). Areas requiring substantial improvements were priority-setting during *research question identification*, which had the lowest mean score of 2.12. This was followed by *promoting use of postgraduate research products*, tying at mean scores of 2.28 each. The domain *knowledge transfer of postgraduate research products* had an above-average mean score of 2.75.

**Table 2:** Mean score per domain of postgraduate research support activities at the departmental level

Domains	*N* = 185	Mean (SD)
Research question identification (priority-setting)	139	2.12 (1.08)
Proposal development for postgraduate research project	180	3.53 (0.97)
Knowledge transfer of postgraduate research products	158	2.75 (0.88)
Promoting use of postgraduate research products	180	2.28 (1.03)

Range, levels 1–5; level 1—the situation is quite unfavourable and/or there is a dire need for intervention; level 5—the situation is good and needs no intervention

### Mean score and standard deviation for each statement in domain

#### Proposal development

Overall, students reported good support from their supervisors (Table 3). Postgraduate students easily accessed mentors during proposal development, which scored highest in the whole assessments with a mean of 4.10 (SD = 1.13). In this subsection, considering the “PICO” approach in research question development had the lowest mean score of 2.64 (SD = 1.58). PICO is a framework for research question development that stands for population, intervention, control and outcome elements.

**Table 3:** Mean score and standard deviation for each statement in domain

Domain 1: Research question development at departmental and individual effort level, *N* = 139 (SD)			
My unit or department or faculty:		Mean (SD)	
(a)	Develops a list of priority questions for further research	2.05 (1.26)	
(b)	Organizes research question identification meetings or seminars with research producers	2.35 (1.43)	
(c)	Holds regular meetings with health research users	1.96 (1.29)	
During the process of identifying my research question, I:		Yes	No
(d)	Identified a health-related problem during the course of my work	127 (91.4)	12 (8.6)
(e)	Identified a gap in literature through reading a systematic review	90 (64.8)	49 (35.7)
(f)	Identified a gap in literature through reading a single study article	27 (19.4)	112 (80.6)
(g)	Consulted my colleagues to identify a suitable research question	72 (51.8)	67 (48.2)
(h)	Consulted my research mentor or potential supervisor	100 (71.9)	39 (28.1)
(i)	Identified a question from an existing project where I am affiliated	30 (21.6)	109 (78.4)
(j)	Identified a question from an existing public list of priority questions	36 (25.9)	103 (74.1)
(k)	Identified a question through interaction with decision-makers	24 (17.3)	115 (82.7)
Domain 2: Proposal development. In conducting your research project, which of the following applies to your department or faculty? *N* = 180 (SD)			
(a)	Assigned a supervisor/mentor	3.82 (1.56)	
(b)	Accessed university guidelines/resources	3.98 (1.28)	
(c)	Accessed my supervisor/mentor easily	4.10 (1.13)	
(d)	Accessed alternative online resources	3.12 (1.59)	
(e)	Considered the PICO approach in research question development	2.64 (1.58)	
Domain 3: Knowledge transfer. In conducting your research project, which of the following applies to your department or faculty? *N* = 158 (SD)			
(a)	There is a process that determines which research results can be transferred to the target audiences	2.67 (1.44)	
(b)	Research results are peer-reviewed prior to knowledge dissemination or transfer	3.35 (1.38)	
(c)	Knowledge transfer and utilization of research results exist in the general programme of research methodology training	2.92 (1.47)	
(d)	I am familiar with the topic of knowledge translation and how to perform it	2.91 (1.39)	
(e)	I am familiar with converting research results into actionable messages appropriate to the target audience	3.08 (1.32)	
(f)	I am familiar with communication skills for knowledge transfer	3.15 (1.33)	
(g)	A list of potential (postgraduate student) research result users is prepared for each research project	2.18 (1.27)	
(h)	The necessary structures and/or manpower are available for strengthening knowledge transfer in our department	2.65 (1.39)	
(i)	The framework of students research projects’ final reports is such that decision-makers can easily point out the actionable message	2.85 (1.24)	
(j)	Student researchers can provide the results of their research through the web and/or electronic banks	2.75 (1.43)	
(k)	There is regular communications with media and target for transfer of research-based evidence	1.81 (1.15)	
(l)	Intellectual property rights exist which support researchers who help disseminate research results prior to their publication in journals	2.21 (1.32)	
(m)	There are criteria for evaluation of researchers’ knowledge transfer activities in our department	2.58 (1.46)	
(n)	Evidence-based medicine/decision-making is among the subjects in our department	3.44 (1.41)	
Domain 4: Promoting use. In conducting your research project, which of the following applies to your unit or department or faculty? *N* = 180 (SD)			
(a)	Systematic reviews or policy briefs or clinical guidelines or technical reports, etc., that strengthen evidence-based decision-making are produced in my department	2.76 (1.44)	
(b)	Postgraduate student researchers participate in technical committees that help in decision-making	1.84 (1.28)	
(c)	We send decision-makers reminders to follow the (postgraduate) research results that we have previously sent them	1.68 (1.14)	
(d)	Education programmes such as “evidence-based medicine” or “evidence-based decision-making” for service providers and/or managers is among the activities in our department	2.84 (1.45)	

### Engagement for priority-setting and knowledge transfer

A further dissection of the specific areas of concern revealed that eight areas were of priority. In terms of research question development, these assessed whether the department (a) developed a list of priority questions for further research, with a below-average mean score of 2.05 (SD = 1.26); (b) organized research question identification meetings or seminars with research producers, mean score 2.35 (SD = 1.43), and (c) held regular meetings with health research users or decision-makers, with a mean score of 1.96 (SD = 1.29). Indeed, very few postgraduate students stated that they interacted with decision-makers during research question identification (17.3%) or identified research questions from an existing public list of priority questions (25.9%).

Across the four domains, the interaction between postgraduate researchers and research purveyors or users had the lowest mean scores. Specific areas were whether the department (d) mapped stakeholders by preparing a list of potential research results users for each postgraduate research project (2.18, SD = 1.27); (e) encouraged postgraduate student researchers to participate in technical committees of the Uganda Ministry of Health or districts or hospitals or even nongovernmental organizations (NGOs) that help in decision-making (1.84, SD = 1.28); (f) saw the need for regular communications with media and target for transfer of research-based evidence (1.81, SD = 1.15); and (g) sent decision-makers reminders to follow the (postgraduate) research results that we have previously sent them (1.68, SD = 1.14).

### Intellectual property

Notably, whether (h) intellectual property rights existed which support researchers who help disseminate research results prior to their publication in journals scored low as well, 2.21 (SD = 1.32).

### Other areas that had suboptimal, above-average mean scores

Finally, there are important areas that need attention despite posting above-average mean scores. These were predominantly in the “knowledge transfer” and “promoting use of knowledge” domains. Teaching evidence-based medicine and the peer review process were ranked highly under the knowledge transfer domain, with means scores 3.44 (SD = 1.41) and 3.35 (SD = 1.38), respectively. Still, the availability of skilled manpower, curriculum content and an appropriate activity schedule for knowledge transfer at department level had suboptimal mean scores between 2.58 (SD = 1.46) for “criteria for evaluation of researchers’ knowledge transfer activities” and 2.92 (SD = 1.47) for “knowledge transfer and utilization of research results exist in the general programme of research methodology training”, respectively, in Table 2.

## Discussion

### Principal findings

In sum, this study highlights that support mechanisms exist for research production by postgraduate students in four Ugandan universities. On the one hand, the most highly rated was the easy access to supervisors and mentors during proposal development. On the other hand, there were eight areas that require improvement around research priority-setting and communication. Specifically, interaction between research users, that is, decision-makers and the media fraternity were limited at the priority-setting stage and results dissemination. Also, awareness of intellectual property rights was low among the postgraduate student researchers.

### Findings in relation to other studies

Indeed, the findings of this study were unique, yet consistent with the evidence base on research support mechanisms for students world over. First, this study found that students were strongly supported to develop their proposals. Previous studies demonstrated that students would be more productive in research if their supervisors or mentors supported their projects in practical ways [16, 19]. The support mechanisms included manuscript writing seminars, workshops, co-supervision or allocation of protected time to concentrate on manuscript writing and funding by specific targeted grants [4, 5].

Secondly, the unique discoveries were specific to the suboptimal engagement activities with end users of research, decision-makers and the media fraternity. Previous studies documented that an integrated approach in this three-way interaction, research producers, purveyors and consumers, increases the uptake and use of research evidence [27, 30]. There is hardly any evidence out there capturing views about graduate students’ research support and engagement with end users of this research, which this study has provided for the first time. Noteworthy, our earlier work documented that, indeed, findings from postgraduate students’ theses informed decision-making documents of international agencies, including WHO [24, 26]. Taken together, it is probable that students strive to push their findings to the decision-making table via publications rather than active engagement with end users of their research.

Obviously, intellectual property protection is an important interest for researchers, students, policy-makers and practitioners [28]. However, as with previous studies, postgraduate students were not properly informed or taught about the concept of intellectual property protection [1]. Intellectual property rights safeguard discoveries and give lawful control to the discoverer in the use of their discoveries. Noteworthy, from our earlier work, only one in five postgraduate students at Makerere University had their thesis published [25]. This coupled with the type of research conducted (less drug, vaccine or device discovery and more observational studies) [25] may suggest a lower demand for intellectual property rights information [11].

### Study limitations and strengths

This study had important strengths, including novelty. This is the first study in Uganda that has documented the views of postgraduate students about how they are supported to produce their research projects. Further, this is the first study globally to interrogate postgraduate students’ engagement activities with research users. Hence, this study identified a key area for improvement: engagement with decision-makers and the media for research priorities and research communication beyond scientific publications. In addition, the study had a diverse sample of students from four universities in the public and private education sectors, making the findings more representative.

Despite these interesting results, there were shortcomings. One, qualitative interviews of postgraduate students would have strengthened these findings with unique aspects of their lived experiences. Two, the views of the supervisors and mentors were not captured; hence, the findings were short of a comprehensive outlook at identifying support barriers such as university staffing capacity challenges. Three, the study did not compare performance across departments or schools or universities and instead lumped up the findings, as pre-written in the protocol approved by the ethics committee. Such a purpose would rather identify bottlenecks and share in appropriate private forums with the view of strengthening these, than promoting interdepartmental or university competition. Finally, there were substantial non-responses in some sub-questions that could have affected overall scores.

### Implications for university policy change

Clearly, the findings suggest a number of policy options. Obvious benefits would accrue from creating opportunities for decision-makers to input into the priority-setting process for postgraduate student research. Oftentimes decision-makers do not know where to access quality research. Thus, providing for time in the research curriculum or establishing bridges to existing platforms where students interact with decision-makers is one of the many cost-efficient approaches. Examples of such low-hanging fruit are technical working groups at the Ministry of Health, or national disease control programmes (AIDS, tuberculosis [TB], malaria), district health teams or health-related NGOs.

Beyond that, sharing existing research priority lists or regularly refining and updating them would be important in research question identification. Also, rewarding such engagement in the postgraduate research project assessment scheme would incentivize postgraduate students to demonstrate how they engaged decision-makers at various levels, including hospitals and district health systems.

An area for attention in postgraduate research is in research communication. *“…You don’t really understand something unless you can explain it to your grandmother…”*, is a quote attributed to the eminent scientist Albert Einstein [14]. In many ways, this “grandmother” metaphor would represent the general public in society. Media professionals are the bridge through which Ugandan researchers would reach the community [17]. This interaction would be achieved at priority-setting and results communication workshops specifically designed for science journalists and postgraduate students in medical fields. Relevant departments that teach journalism, media and communication studies in these Ugandan universities could collaborate with medical and public health schools to enhance science communication.

### Implications for future research

Future research should obtain views of supervisors and mentors using qualitative research on the barriers, facilitators and solutions to support mechanisms for postgraduate students in Uganda. Secondly, comparison of different departments or universities’ performance would be enlightening and should go through the institutional research administrative approval processes for legitimacy. Third, prospective assessments of deliberate interaction platforms between postgraduate students and research users, decision-makers and the media would be informative.

## Conclusions

This study reports that existing research support mechanisms for postgraduate students in Uganda encourage access to supervisors and mentors, particularly during proposal development. Secondly, postgraduate students’ engagement with research users was limited, as was their awareness about intellectual property rights. Views of supervisors and mentors were not captured, a subject for future research.

## Data Availability

The lead author does wish to share the data after completion of his doctoral project.

## Abbreviations

AIDS: Acquired immune deficiency syndrome
KT: Knowledge translation
MakCHS: College of Health Sciences, Makerere University
m-SATORI: Modified self-assessment tool for research institutes
MUST: Mbarara University of Science and Technology
NGO: Nongovernmental organization
SATORI: Self-assessment tool for research institutes
TB: Tuberculosis
TUMS: Tehran University of Medical Sciences
UCU: Uganda Christian University, Mukono
UMU: Uganda Martyrs University, Nkozi
